# Elevated spondin-2 expression correlates with progression and prognosis in gastric cancer

**DOI:** 10.18632/oncotarget.14423

**Published:** 2017-01-02

**Authors:** Chuan Jin, Jin-Rong Lin, Lei Ma, Ye Song, Yan-Xia Shi, Peng Jiang, Ye Dong, Xiao-Shan Li

**Affiliations:** ^1^ Department of Medical Oncology, Cancer Center of Guangzhou Medical University, Guangzhou, 510095, Guangdong, China

**Keywords:** spondin-2, gastric cancer, aggressiveness, prognosis

## Abstract

The spondin-2 correlated with tumor progression in many malignancies. However, the role of spondin-2 in gastric cancer has not been thoroughly elucidated. Spondin-2 and matrix metallopeptidase 9 (MMP-9) expression was detected by immunohistochemistry in 174 gastric carcinoma tissues. The relationship between the expression of spondin-2 and MMP-9, clinicopathological/prognostic value in gastric cancer was examined. Spondin-2 was significantly higher in gastric cancer than that in adjacent non-tumorous tissues. Spondin-2 overexpression was significantly associated with well differentiation, depth of invasion, lymph node metastasis, and advanced TNM stages. The expression levels of spondin-2 were increasing in both prominent serosal invasion group and lymph node metastasis group. In addition, spondin-2 was positively correlated with MMP-9 among 174 gastric cancer samples. In univariate and multivariate analyses, spondin-2 was an independent prognostic factor for both recurrence-free survival (RFS) and overall survival (OS). Moreover, spondin-2 overexpression was associated with poor prognosis in patients with gastric cancer in different risk groups. In conclusion, Spondin-2 overexpression contributes to tumor aggressiveness and prognosis, and could be a promising target for prognostic prediction in gastric cancer patients.

## INTRODUCTION

Gastric cancer is an aggressively invasive tumor, and one of the most common lethal cancers worldwide [1, 2]. The incidence of gastric cancer in China and some East Asia countries is much higher than that in America or Europe countries [3]. Surgical resection may successfully treat gastric cancer in the early stage of disease [4]. However, due to atypical symptoms at the early stage, over 80% of patients with gastric cancer were diagnosed at an advanced stage, which usually indicates a poor prognosis [5]. In addition, even the addition of novel, molecularly targeted therapies to standard chemotherapy provide only a limited survival benefit [6]. Increasing numbers of biomarkers have been reported that are associated with different types of cancer [6]. Therefore, the identification of novel biomarkers to predict prognosis is important to guide the treatment of patients with gastric cancer.

Spondin-2 (SPON2, Mindin, DIL-1) belongs to the F-spondin family of secreted extracellular matrix proteins [7]. The members of mindin-F-spondin family have three domains: FS1 (for F-spondin), FS2 and thrombospondin type 1 repeats. Spondin-2 has multiple biological functions, including triggering the innate immune response, recruiting inflammatory cells and developing neurons [8, 9]. Recently, it has been reported that increased spondin-2 gene and protein expression was observed in ovarian cancer [10, 11], liver cancer [12, 13], prostate cancer [14, 15] and pancreatic cancer [16]. The expression of Spondin-2 in gastric cancer tissue is higher than that in adjacent non-tumorous tissues [17]. However, the role of Spondin-2 expression on the prognosis of patients with gastric cancer remains unclear. Therefore, the purpose of this study is to evaluate whether Spondin-2 expression is associated with clinicopathological parameters and prognosis of gastric cancer.

## RESULTS

### The association of spondin-2 with clinicopathological variables

**Figure 1:**
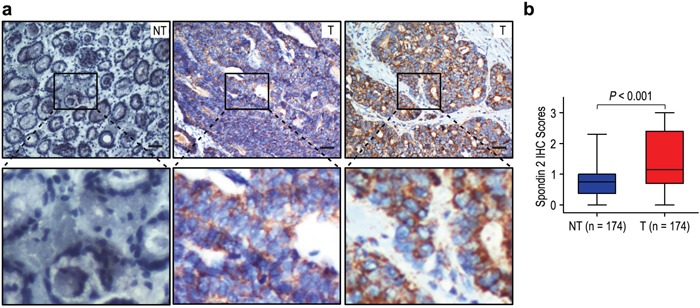
Spondin-2 was significantly up-regulated in gastric cancer **a**. IHC assays of spondin-2 expression in 174 paired gastric cancer samples and adjacent non-tumorous tissues. The upper left panel represents low spondin-2 expression in adjacent non-tumorous tissues. The upper middle and right panel represents low and high spondin-2 expression in gastric cancer. Lower panels represent magnified pictures of boxed area in the corresponding upper panels. The scale bar represents 50 μm. **b**. Spondin-2 expression levels were compared with gastric cancer and adjacent non-tumorous specimens.

To investigate the biological significance of Spondin-2 in gastric cancer, we detected the expression of Spondin-2 in 174 gastric cancer tissues (tumor and matched adjacent non-tumorous tissues) by immunohistochemistry. Spondin-2 staining mainly located in cytoplasm of tumor cells (Figure 1a). Overexpression of Spondin-2 was observed in 87 of 174 (50.0%) of gastric cancer samples, compared with 34/174 (19.5%) in adjacent non-tumorous tissues (*P* < 0.001; Figure 1b). The results suggested that Spondin-2 expression was significantly higher in gastric cancer than that in adjacent non-tumorous tissues.

To elucidate the functions of Spondin-2 in gastric cancer, we analyzed spondin-2 status in 174 gastric cancer specimens with other widely recognized clinicopathologic parameters (Table 1). Overexpression of spondin-2 in gastric cancer was significantly associated with well differentiation (*P* = 0.015), depth of invasion (*P* < 0.001), lymph node metastasis (*P* = 0.001), and advanced TNM stages (*P* = 0.004). However, spondin-2 expression displayed no association with gender, age, tumor size and tumor site (*P* > 0.05) (Table 1). The association of clinicopathologic parameters with spondin-2 expression in adjacent non-tumorous tissues and MMP-9 expression in gastric cancer was showed in Supplementary Table 1. Notably, the association of spondin-2 expression with prominent serosal invasion and lymph node metastasis positivity suggested a potential role of spondin-2 in increased invasion and metastasis of gastric cancer.

**Table 1:** Clinicopathologic correlation of spondin-2 expression in 174 gastric cancer

Characteristics	No. of patients	spondin-2 expression (%)		P-value
		Low	High	
Gender				
Male	113	51 (45.1%)	62 (54.9%)	
Female	61	36 (59.0%)	25 (41.0%)	0.081
Age (years)				
≤ 60	104	49 (47.1%)	55 (52.9%)	
> 60	70	38 (54.3%)	32 (45.7%)	0.354
Size (cm)				
≤ 5.0	110	61 (55.5%)	49 (44.5%)	
> 5.0	64	26 (40.6%)	38 (59.4%)	0.059
Tumor site				
Upper	78	33 (42.3%)	45 (57.7%)	
Middle/Lower	96	54 (56.3%)	42 (43.7%)	0.067
Differentiation				
Well/Moderate	82	33 (40.2%)	49 (59.8%)	
Poor	92	54 (58.7%)	38 (41.3%)	0.015
Depth of invasion				
T1/T2	66	45 (68.2%)	21 (31.8%)	
T3/T4	108	42 (38.9%)	66 (61.1%)	< 0.001
Lymph node metastasis				
Negative	47	33 (70.2%)	14 (29.8%)	
Positive	127	54 (42.5%)	73 (57.5%)	0.001
TNM stages				
I/II	58	38 (65.5%)	20 (34.5%)	
III/IV	116	49 (42.2%)	67 (57.8%)	0.004

### Impact of spondin-2 overexpression on invasion and metastasis in gastric cancer

**Figure 2:**
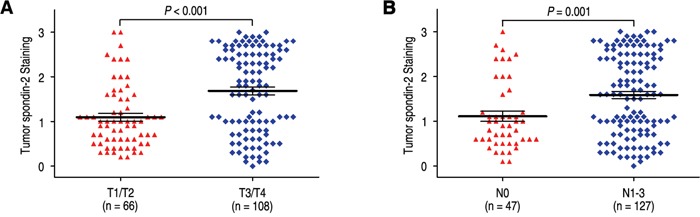
Comparsion of spondin-2 expression by depth of invasion and lymph node metastasis Spondin-2 expression is markedly increased both prominent serosal invasion group **A**. and lymph node metastasis group **B**.

**Figure 3:**
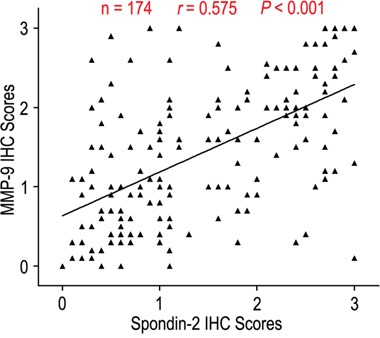
Spondin-2 and MMP-9 protein levels correlated in 174 gastric cancer tissues The scale bar represents 100 μm.

To further explore the significance of spondin-2 expression on aggressiveness in gastric cancer, we firstly discussed the relationship of spondin-2 expression with depth invasion and lymph node metastasis. The rates of spondin-2 overexpression were 61.1% and 57.5% in the T3/T4 group and N1-3 group, while there were only 31.8% and 29.8% in T1/T2 and N0 groups (*P* < 0.001 and *P* = 0.001, respectively) (Table 1). In addition, the expression levels of spondin-2 were higher in T3/T4 and N1-3 compared with those in T1/T2 and N0 (*P* < 0.001 and *P* = 0.001, respectively) (Figure 2). It has been reported that MMP-9 was associated with aggressiveness in gastric cancer. Therefore, we also evaluated the correlation of Spondin-2 and MMP-9 expression in gastric cancer patients and found that Spondin-2 was positively correlated with MMP-9 expression in 174 gastric carcinoma tissues (Figure 3, r = 0.575, *P* < 0.001).

### Correlation between spondin-2 expression and prognosis

**Figure 4:**
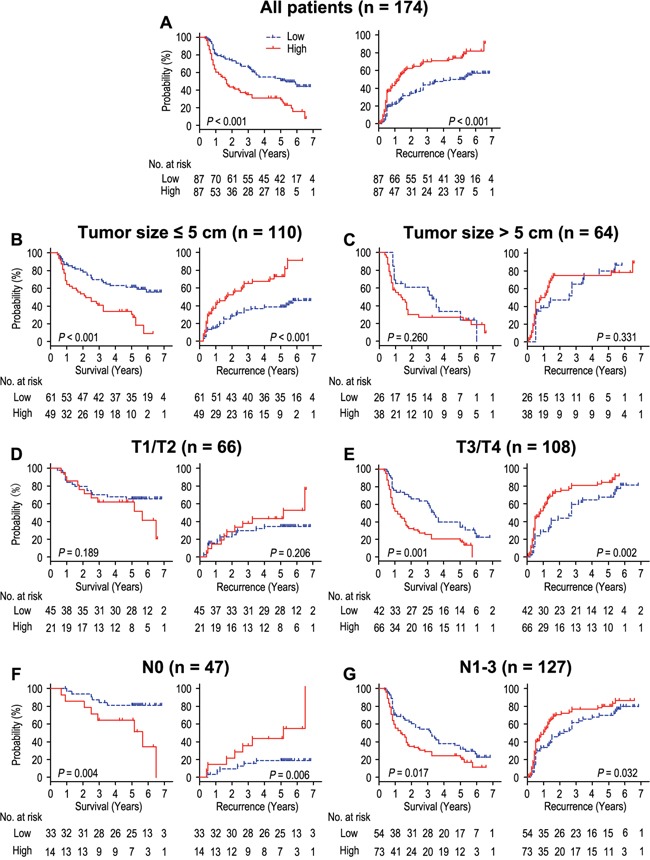
OS and RFS are shown for gastric cancer patients All patients were stratified according to tumor size, depth of invasion and lymph node metastasis. Kaplan-Meier survival estimates and log-rank tests were used to analyze the prognosis of spondin-2 expression in all patients **A**. and each subgroup **B-G**.

The Kaplan-Meier survival analysis revealed that OS and RFS with high spondin-2 expression were significantly shorter than those with low spondin-2 expression (*P* < 0.001 and *P* < 0.001, respectively) (Figure 4A). The postoperative median RFS and OS were 20.5 months and 30.0 months, respectively. The postoperative median RFS and OS of patients with high spondin-2 expression were 13.0 months and 19.0 months, while there were 41.0 months and 44.0 months of those with low spondin-2 expression. For patients with high spondin-2 expression, the cumulative1-, 3- and 5-year survival rates were 64.0%, 34.7% and 30.9%, respectively, which was significantly lower than those for patients with low spondin-2 expression (81.4%, 66.7% and 53.0%, respectively) (Table 2). In addition, in order to evaluate the prognostic value of Spondin-2 in different subgroups, patients were stratified according to tumor size (Figure 4B, 4C), depth of invasion (Figure 4D, 4E), and lymph node metastasis (Figure 4F, 4G). The overexpression of Spondin-2 remained its prognostic value in predicting shorter OS and RFS in the subgroup of lymph node metastasis. For the subgroups of smaller tumor size and T3/T4 patients, significant correlations were found between Spondin-2 status and OS (*P* < 0.001 and *P* = 0.001, respectively) and RFS (*P* < 0.001 and *P* = 0.002, respectively). Spondin-2 had no prognostic value regarding OS or RFS for patients with larger tumor size and T1/T2 (all *P* > 0.05). Therefore, it suggests that Spondin-2 may serve as a potential prognostic biomarker for gastric cancer patients in different risk groups.

**Table 2:** Predictive variables for recurrence-free survival and overall survival of 174 patients with gastric cancer

Variables	No. of patients	RFS rate (%)		P-value	OS rate (%)		P-value
		3 y	5 y		3 y	5 y	
Gender							
Male	113	42.7	36.1		48.4	41.1	
Female	61	44.3	41.0	0.575	55.7	44.2	0.475
Age (years)							
≤ 60	104	47.3	44.0		52.2	46.7	
> 60	70	37.4	29.6	0.255	49.5	35.8	0.188
Size (cm)							
≤ 5.0	110	51.5	46.5		57.1	49.2	
> 5.0	64	28.6	22.9	< 0.001	40.5	29.3	0.001
Tumor site							
Upper	78	34.3	28.1		45.5	33.8	
Middle / Lower	96	50.1	45.5	0.023	55.3	48.5	0.031
Differentiation							
Well / Moderate	82	41.9	35.1		48.4	40.6	
Poor	92	44.2	40.5	0.823	53.4	43.4	0.584
Depth of invasion							
T1/T2	66	67.5	62.6		67.5	64.1	
T3/T4	108	27.8	22.4	< 0.001	40.7	28.1	< 0.001
Lymph node metastasis							
Negative	47	78.4	73.8		80.5	76.0	
Positive	127	29.7	24.2	< 0.001	39.7	29.0	< 0.001
Spondin-2 protein expression							
Low	87	56.1	49.8		67.1	53.4	
High	87	30.1	25.9	< 0.001	34.7	30.9	< 0.001

To further confirm the effect of Spondin-2 status on RFS and OS in gastric cancer, we firstly performed univariate analysis of traditional clinicopathologic parameters for prognosis. Significant variables in the RFS and OS analysis included spondin-2 overexpression (*P* < 0.001 and *P* < 0.001, respectively), larger tumor size (*P* < 0.001 and *P* = 0.001, respectively), tumor site (*P* = 0.023 and *P* = 0.031, respectively), prominent serosal invasion (*P* < 0.001 and *P* < 0.001, respectively) and lymph node metastasis (*P* < 0.001 and *P* < 0.001, respectively) (Table 2). Moreover, to evaluate the independent impact of spondin-2 overexpression on RFS and OS, a multivariate Cox regression model adjusted for tumor size, tumor site, depth of invasion, lymph node metastasis and spondin-2 expression was performed. Our results revealed that overexpression of Spondin-2 was almost 1.6 times more likely to suffer from relapse than those with low Spondin-2 expression (HR = 1.600, 95% CI: 1.085-2.359; *P* = 0.018). Furthermore, the patients with high Spondin-2 expression was a poor independent predictor for OS in gastric cancer patients (HR = 1.747, 95% CI: 1.178-2.591; *P* = 0.006). In addition, tumor size, depth of invasion and lymph node metastasis all had independent prognostic value in the multivariate analysis (Table 3).

**Table 3:** Multivariate Cox regression analysis for recurrence-free survival and overall survival in patients with gastric cancer

Variable	RFS (n = 174)		OS (n = 174)	
	Hazard Ratios (95% CI)	*P*-value^a^	Hazard ratios (95% CI)	*P*-value^a^
Tumor size	1.665 (1.131 ∼ 2.450)	0.010	1.654 (1.118 ∼ 2.445)	0.012
Tumor site	0.922 (0.624 ∼ 1.361)	0.683	0.962 (0.644 ∼ 1.436)	0.850
Depth of invasion	1.683 (1.011 ∼ 2.864)	0.043	1.581 (1.002 ∼ 2.715)	0.045
Lymph node metastasis	3.026 (1.567 ∼ 5.846)	0.001	2.916 (1.500 ∼ 5.668)	0.002
Spondin-2 expression	1.600 (1.085 ∼ 2.359)	0.018	1.747 (1.178 ∼ 2.591)	0.006

^a^ Cox proportional hazard model regression.

## DISCUSSION

To our knowlege, the association between spondin-2 overexpression and the prognosis of gastric cancer have not been reported. In the present study, the expression of spondin-2 was investigated in 174 gastric carcinoma samples by IHC. We found that spondin-2 was up-regulated in gastric cancer tissues compared with adjacent non-tumorous tissues. In addition, overexpression of spondin-2 was significantly associated with well differentiation, depth of invasion, lymph node metastasis, and advanced TNM stages in gastric cancer. Moreover, the Kaplan-Meier survival analysis revealed that the patients with high spondin-2 expression had poorer survival times (RFS and OS) than those with low spondin-2 expression. The prognostic value of spondin-2 in different subgroups based on tumor size, depth of invasion and lymph node metastasis was also estimated, which appears that spondin-2 may serve as a prognostic factor for gastric cancer patients in different risk groups. Furthermore, the multivariate Cox model analysis indicated that spondin-2 up-regulation was an independent factor for poor RFS as well as OS in gastric cancer. This finding concludes that spondin-2 correlate to tumor prognosis and that spondin-2 could be a potential prognostic factor of gastric cancer. The results of this study were consistent with previously reported results. In several investigations, it has been shown that the abnormal expression of spondin-2 in cancer cells is associated with tumor progression. Zhang Q et al.[18] reported that the expression of spondin-2 was increased in colorectal cancer (CRC) and was significantly associated with CRC stage, T stage, M stage and Dukes stage. Moreover, spondin-2 could be an independent diagnostic and prognostic biomarker of colon cancer. Additionally, Lucarelli G et al.[19] found that spondin-2 levels were significantly higher in patients with prostate cancer than in healthy individuals. Spondin-2 had better diagnostic performance than serum sarcosine, and percent free-to-total and total prostate specific antigen. Our findings and previous observations strongly suggest that spondin-2 overexpression is involved in the tumor progression and may work as a prognostic factor for gastric cancer patients.

The degradation of extracellular matrix (ECM) is a signal for the beginning of invasion and metastasis, and matrix metalloproteases (MMPs) are important molecules involved in ECM degradation during invasion and metastasis [20]. Chu et al.[21] showed that up-regulation of MMP-9 was positively associated with depth of invasion and lymph node metastasis in gastric cancer, and the patients with high MMP-9 expression had worse prognosis than those with low MMP-9 expression. Moreover, Zhao et al.[22] reported that down-regulation of MMP-9 by RNA interference was able to suppress the MMP-9 expression, inhibit tumor cell growth and invasion of SGC7901 gastric cancer *in vitro* and *in vivo*. Furthermore, Liao CH et al.[12] showed that spondin-2, which was regulated by thyroid hormone 3, 3′, 5-triiodo-L-thyronine, had an important role in cell invasion, cell migration and tumor progression in hepatocellular carcinomas. Our results revealed the expression of spondin-2 was up-regulation in patients with depth of invasion (T3/T4) and lymph node metastasis (N1-3) indicating higher invasive and metastasizing activity in spondin-2 high-expression cancer cells. In addition, spondin-2 was positively correlated with MMP-9 protein expression in gastric cancer. Collectively, spondin-2 expression in gastric cancer promoting tumor progression indicates that spondin-2 could be a feasible target in cancer therapy.

## CONCLUSION

In summary, we proved that overexpression of spondin-2 may have positive role in tumor invasion, lymph node metastasis and prognosis, and could be a promising biomarker for prognostic prediction in gastric cancer. Detection the spondin-2 expression may help to identify the gastric cancer patients with high-risk factors and thus aid the selection of appropriate therapies. Further studies are warranted to more firmly establish this supposition.

## MATERIALS AND METHODS

### Patients and specimens

This study was approved by the Institutional Review Board and Human Ethics Committee of Cancer Center of Guangzhou Medical University. Written consent for using the samples for research purposes was obtained from all patients prior to surgery.

All the gastric cancer samples and adjacent non-tumorous gastric tissues were obtained from gastrectomy specimens of 174 patients from the pathology department, the Cancer Center of Guangzhou Medical University (Guangzhou, China). All the operations were performed between January 2006 and October 2008. Patient diagnosis was established pathologically, and none of the patients had received chemotherapy or radiotherapy prior to surgery. The cases were selected consecutively on the basis of availability of resection tissues and follow-up data. There were 113 males and 61 females (median age, 58.0 years; range, 21-83 years). Relevant clinical pathologic features were all collected from the patients’ files. Tumor stage was classified according to the 7th Union International Cancer Control (UICC) TNM staging system [23].

### Immunohistochemistry staining

A total of 174 gastric carcinoma samples and their adjacent non-tumorous tissues were detected by immunohistochemistry (IHC). Formalin-fixed, paraffin-embedded specimens from consenting patients were cut in 4 μm sections. Slides were baked at 55°C for 1 h, deparaffinized with xylene and rehydrated using an alcohol gradient. The tissue slides were then treated with 3% hydrogen peroxide in methanol for 10 min to quench endogenous peroxidase activity, and the antigens were retrieved in 0.01 M sodium citrate buffer (pH 6.0) using microwave oven. After 15 min of preincubation in 10% normal goat serum to prevent nonspecific staining, the samples were incubated overnight using a primary antibody, either anti-spondin-2 (Abcam, #ab187920, UK, dilution 1:200) or anti-MMP-9 (Abcam, #ab38898, UK, dilution 1:200), in a humidified container at 4°C. The tissue slides were treated with a non-biotin horseradish-peroxidase detection system according to the manufacturer’s instructions (Gene Tech). Assessments of the staining were scored by two experienced pathologists blinded to the patients’ identity and clinical status. In discrepant cases, a pathologist reviewed the cases and reached the consensus.

Both the extent and intensity of immunostaining were taken into consideration when analyzing the data. The intensity of staining was scored from 0 to 3, and the extent of staining was scored from 0% to 100%. The final quantitation of each staining was obtained by multiplying the two scores. Spondin-2 expression was classified as high expression if the score was higher than the median score of 1.1, if the score was 1.1 or less, the case was classified as low expression. MMP-9 expression was considered high if the score was higher than 1.4 (Supplementary Figure 1).

### Follow-up

All the patients had follow-up records for more than 5 years. The follow-up deadline was October 2015. Patients had follow-up appointments every 3 months for the first 3 years after surgery, every 6 months for the next 2 years, and yearly thereafter. The median follow-up period was 30.0 months (range, 3.0 – 82.0 months) in 174 patients. Recurrence were confirmed by tumor markers levels including CEA, AFP, CA199, CA125 and CA724, B-type ultrasonic inspection every 3 moths, and computed tomography (CT) or magnetic resonance imaging (MRI) every 6 months after gastrectomy. The main causes of death were gastric cancer recurrence. Overall survival (OS) was defined as from the date of surgery to the date of death or last follow-up. Recurrence-free survival (RFS) was measured from the date of surgery until the date of relapse, metastasis, or last follow-up.

### Statistical analysis

All statistical analyses were carried out using SPSS software (version 16.0; Chicago, IL, USA). Interdependence between spondin-2 expression and clinical data was calculated using the chi-square test, and displayed in cross-tables. Correlation of spondin-2 with MMP-9 staining scores was calculated by Pearson χ2 test. Survival curves were generated using the Kaplan-Meier method, and the univariate nanlyses were estimated by the log-rank test. The Cox multivariate proportional hazards regression model was used to determine the independent factors that influence survival and recurrence based on the investigated variables. All reported *P* values were two-sided and *P* < 0.05 was considered statistically significant.

## SUPPLEMENTARY FIGURE AND TABLE



## Abbreviations

MMP-9: matrix metallopeptidase 9
UICC: Union International Cancer Control
IHC: immunohistochemistry
RFS: recurrence-free survival
OS: overall survival
