# Supplementary material for: Elevated spondin-2 expression correlates with progression and prognosis in gastric cancer
Source: Oncotarget. 2017 Jan 2;8(6):10416–24. doi: 10.18632/oncotarget.14423 (PMC5354668; doi:10.18632/oncotarget.14423)
Supplement: Supplementary file 1 [file oncotarget-08-10416-s001.pdf]

Elevated spondin-2 expression correlates with progression and prognosis in gastric cancer

SUPPLEMENTARY FIGURE AND TABLE

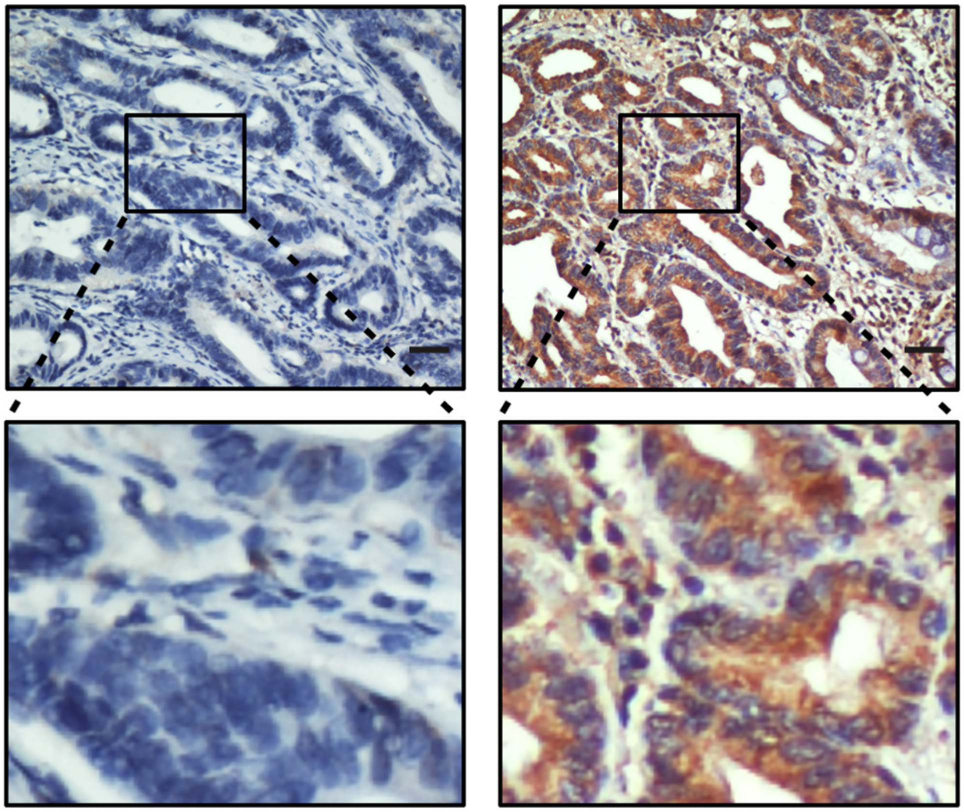

Supplementary Figure 1: MMP-9 was significantly up-regulated in gastric cancer. The upper left and right panel represents low and high MMP-9 expression in gastric cancer tissues. Lower panels represent magnified pictures of boxed area in the corresponding upper panels. The scale bar represents 50 μm.

Supplementary Table 1: Clinicopathologic correlation of spondin-2 and MMP-9 expression in 174 gastric cancer and matched adjacent non-tumorous tissues

Characteristics	No. of patients	adjacent non-tumorous tissues spondin-2 expression (%)		*P*-value	gastric cancer tissues MMP-9 expression (%)		*P*-value
		Low	High		Low	High	
Gender							
Male	113	92 (71.4%)	21 (18.6%)		54 (47.8%)	59 (52.2%)	
Female	61	48 (78.7%)	13 (21.3%)	0.665	32 (52.5%)	29 (47.5%)	0.556
Age (years)							
≤ 60	104	85 (81.7%)	19 (18.3%)		52 (50.0%)	52 (50.0%)	
> 60	70	55 (78.6%)	15 (21.4%)	0.606	34 (48.6%)	36 (51.4%)	0.853
Size (cm)							
≤ 5.0	110	92 (82.9%)	18 (17.1%)		57 (62.0%)	53 (38.0%)	
> 5.0	64	48 (75.0%)	16 (25.0%)	0.166	29 (45.3%)	35 (54.7%)	0.408
Tumor site							
Upper	78	59 (75.6%)	19 (24.4%)		38 (48.7%)	40 (51.3%)	
Middle/ Lower	96	81 (84.4%)	15 (15.6%)	0.148	48 (50.0%)	48 (50.0%)	0.866
Differentiation							
Well/ Moderate	82	62 (75.6%)	20 (24.4%)		37 (45.1%)	45 (54.9%)	
Poor	92	78 (84.8%)	14 (15.2%)	0.128	49 (53.3%)	43 (46.7%)	0.284
Depth of invasion							
T1/T2	66	60 (90.9%)	6 (9.1%)		42 (63.6%)	24 (36.4%)	
T3/T4	108	80 (74.1%)	28 (25.9%)	0.007	44 (40.7%)	64 (59.3%)	0.003
Lymph node metastasis							
Negative	47	42 (89.4%)	5 (10.6%)		28 (59.6%)	19 (40.4%)	
Positive	127	98 (77.2%)	29 (22.8%)	0.072	58 (45.7%)	69 (54.3%)	0.103
TNM stages							
I/II	58	51 (87.9%)	7 (12.1%)		35 (60.3%)	23 (39.7%)	
III/IV	116	89 (76.7%)	27 (23.3%)	0.079	51 (44.0%)	65 (56.0%)	0.042
